# Norovirus Infection in Young Nicaraguan Children Induces Durable and Genotype-Specific Antibody Immunity

**DOI:** 10.3390/v14092053

**Published:** 2022-09-16

**Authors:** Paul D. Brewer-Jensen, Yaoska Reyes, Sylvia Becker-Dreps, Fredman González, Michael L. Mallory, Lester Gutiérrez, Omar Zepeda, Edwing Centeno, Nadja Vielot, Marta Diez-Valcarce, Jan Vinjé, Ralph Baric, Lisa C. Lindesmith, Filemon Bucardo

**Affiliations:** 1 Department of Epidemiology, University of North Carolina at Chapel Hill, Chapel Hill, NC 27599, USA; 2 Department of Microbiology, Faculty of Medical Sciences, National Autonomous University of Nicaragua, León 21000, Nicaragua; 3 Division of Molecular Medicine and Virology, Department of Clinical and Experimental Medicine, Linköping University, SE-581 83 Linköping, Sweden; 4 Department of Family Medicine, University of North Carolina at Chapel Hill, Chapel Hill, NC 27599, USA; 5 Division of Viral Diseases, U.S. Centers for Disease Control and Prevention, Atlanta, GA 30329, USA

**Keywords:** norovirus, immunity, diarrhea, gastroenteritis, children, Nicaragua, blockade antibody

## Abstract

There are significant challenges to the development of a pediatric norovirus vaccine, mainly due to the antigenic diversity among strains infecting young children. Characterizing human norovirus serotypes and understanding norovirus immunity in naïve children would provide key information for designing rational vaccine platforms. In this study, 26 Nicaraguan children experiencing their first norovirus acute gastroenteritis (AGE) episode during the first 18 months of life were investigated. We used a surrogate neutralization assay that measured antibodies blocking the binding of 13 different norovirus virus-like particles (VLPs) to histo-blood group antigens (HBGAs) in pre- and post-infection sera. To assess for asymptomatic norovirus infections, stools from asymptomatic children were collected monthly, screened for norovirus by RT-qPCR and genotyped by sequencing. Seroconversion of an HBGA-blocking antibody matched the infecting genotype in 25 (96%) of the 26 children. A subset of 13 (50%) and 4 (15%) of the 26 children experienced monotypic GII and GI seroconversion, respectively, strongly suggesting a type-specific response in naïve children, and 9 (35%) showed multitypic seroconversion. The most frequent pairing in multitypic seroconversion (8/12) were GII.4 Sydney and GII.12 noroviruses, both co-circulating at the time. Blocking antibody titers to these two genotypes did not correlate with each other, suggesting multiple exposure rather than cross-reactivity between genotypes. In addition, GII titers remained consistent for at least 19 months post-infection, demonstrating durable immunity. In conclusion, the first natural norovirus gastroenteritis episodes in these young children were dominated by a limited number of genotypes and induced responses of antibodies blocking binding of norovirus VLPs in a genotype-specific manner, suggesting that an effective pediatric norovirus vaccine likely needs to be multivalent and include globally dominant genotypes. The duration of protection from natural infections provides optimism for pediatric norovirus vaccines administered early in life.

## 1. Introduction

Norovirus is among the most common causes of acute viral gastroenteritis globally, detected in 18% of AGE episodes, with a similar prevalence in high- and low-income countries [1,2,3,4]. Birth cohort studies have shown that up to 70% of children experience norovirus-associated AGE episodes during the first two years of life [5]. Noroviruses cause approximately 200,000 deaths annually; a high proportion of these deaths occur in children under five years of age in developing countries [6]. This high burden of disease in children argues for the need for an effective pediatric norovirus vaccine.

Norovirus is a non-enveloped single-stranded RNA virus [7] containing three open reading frames (ORF): ORF1 encodes six nonstructural proteins, including the RNA-dependent RNA polymerase (RdRp); ORF2 encodes VP1 (57 kDa), the major capsid protein; and ORF3 encodes VP2, the minor capsid protein [8]. VP1 is a major antigenic determinant and is the basis for norovirus classification. The P2 subdomain is the most exposed region of the VP1 structure [9] and is involved in the interactions with histo-blood group antigens (HBGAs), which are recognized as a possible cellular receptor or binding cofactor [10,11]. The expression of HBGAs defines the Secretor, Lewis and blood group phenotypes; individuals expressing these antigens are more susceptible to the infection with the most common norovirus genotypes compared to individuals who do not express HBGAs (non-secretors) [12,13,14]. The HBGA phenotype varies greatly between different populations, thus epidemiologically dominant strains of norovirus display specificity for glycan motifs present in large fractions of the population [13,14].

Norovirus’s genetic diversity is represented by 48 genotypes distributed into 10 genogroups (G) [15]. Viruses in GI and GII are commonly found in humans, with a single genotype (GII.4) accounting for more than 50% of all reported norovirus infections in children [16]. New genetic variants within GII.4 emerge periodically and cause epidemic waves of acute gastroenteritis globally [17,18,19]. Other genotypes such as GII.2, GII.3, GII.6 and GII.17 have emerged and transiently replaced GII.4 in some geographic areas during specific time frames [16,20,21,22].

A major challenge for norovirus vaccine development is the high genetic and antigenic diversity of this virus [23]. Phylogenetic studies have shown that some genotypes remain genetically static, while others evolve over time [24]. Antigenic analyses of GII.4 variants have demonstrated significant evolutionary change within antibody epitopes between epidemic strains, providing direct evidence that GII.4 noroviruses are undergoing antigenic variation [25]. Some efforts have been made to define serotypes and to investigate patterns of cross-protection within and between genogroups [26,27,28,29], but most of these studies have been conducted on serum from adults which contains highly cross-reactive antibodies (Abs) due to repeated exposures throughout life [28]. In comparison, birth cohort studies provide the opportunity to define serotypes because the history of natural norovirus infections and their impact on the development of immunity can be captured from a naïve state.

In the absence of a widely available in vitro propagation system for human norovirus [30], neutralizing antibody potency is most frequently determined with a surrogate assay that measures the potency of the antibody for blocking the interaction of norovirus virus-like particles (VLPs) with native carbohydrate binding ligands [25]. The “blockade antibody” assay highly correlates with in vitro live virus neutralization and protection from infection in a human intestinal enteroid system [31,32,33]. A clinical trial showed that adults challenged with GII.4 develop Abs that block the binding of norovirus to HBGAs [28], and that HBGA-blocking Abs best correlate with protection [34]. In addition, a human challenge study with the GII.2 Snow Mountain strain elicited a blockade response against the homotypic VLP, and cross-genotype activity was observed in some individuals, but no cross-genogroup activity was observed [35].

While serum blockade Abs from adults show broad responses against multiple genotypes, likely reflecting multiple past norovirus infections, narrow response has been reported in young children [28]. A follow-up study of two children for eight years suggested genotype-specific immunity with a modest level of cross-protection between genotypes [36]. Additional immunological studies with larger sample numbers are needed to investigate humoral immunity in young children experiencing their first norovirus infections to understand how breadth of immunity develops and how long humoral immunity lasts. 

In this study, we investigated the levels, breadth and duration of HBGA-blocking Abs following the first norovirus episodes in young children followed prospectively from birth until three years of age, whose norovirus infection history was well-defined. The blockade Ab response was investigated against a panel of 13 different norovirus VLPs belonging to GI and GII, including two GII.4 variants (Sydney and Den Haag). The findings may have implications for the number of antigens to be considered, as well as the immunization schedules of future pediatric norovirus vaccines.

## 2. Materials and Methods

### 2.1. Study Design

Samples from 26 norovirus-positive children (all secretors [12]) were obtained from a population-based birth cohort of 444 children in León, Nicaragua. Children were enrolled between 12 June 2017 and 31 July 2018 and contacted in their homes weekly to screen for AGE episodes. Eligibility criteria for children in the cohort and the definition of AGE episodes have been described elsewhere [37]. Informed consent was provided by the child’s mother or legal guardian, and the study received approval from the Institutional Review Boards of the University of North Carolina at Chapel Hill (Study #: 16-2079), the National Autonomous University of Nicaragua, León (UNAN-León, Acta No. 45, 2017) and the CDC (project ID: 0900f3eb81c526a7).

### 2.2. Specimen Collection

Stool samples were collected during AGE episodes within two hours of defecation from the participant’s household and transported at 4 °C to the Microbiology Laboratory at UNAN-Leon. A phosphate buffer solution (PBS, pH 7.2) suspension of 10% (wt/vol) was prepared from each sample. In addition, routine (asymptomatic) stools were collected from each child monthly and processed as described above. Blood collections for sera were performed by venipuncture at the following ages: 6 weeks, 5 months, 12 months and every 6 months thereafter until 36 months of age.

### 2.3. Norovirus Detection in AGE Stools

Viral RNA extraction was performed from 140 µL of stool suspension (1:10) using the QIAamp Viral RNA Mini Kit (QIAGEN, Valencia, CA, USA), following the manufacturer’s instructions. Norovirus screening was performed using a quantitative real-time PCR (RT-qPCR) in a duplex format with the AgPath-ID One-Step RT-PCR Kit (Applied Biosystems, Foster City, CA, USA), as described elsewhere [38,39,40]. A value of cycle threshold (Ct) < 37 was considered positive for norovirus GII and a Ct < 35 was considered positive for norovirus GI, as recommended in the Center for Disease Control protocols used in this study. 

### 2.4. Norovirus Detection in Monthly Collected Stools 

Monthly routine stools were collected from each child in the cohort to detect asymptomatic infections. For norovirus screening, five to seven samples from the same child were pooled, with each pool containing 100 µL of each stool suspension (1:10). Viral RNA extraction and RT-qPCR conditions were performed as described above. For any positive pools, individual samples were analyzed separately to determine single or multiple infections.

### 2.5. Nucleotide Sequencing 

Norovirus-positive samples with a low Ct (≤33) were selected to sequence a fragment (570 bp for GII and 579 for GI) spanning the polymerase and capsid gene to determine P and G types, respectively, as described elsewhere [41]. Sequencing was performed by Macrogen Inc. (Rockville, MD, USA) and genotypes were defined by using https://calicivirustypingtool.cdc.gov (accessed on 10 August 2022) [42].

### 2.6. Virus-like Particle (VLP) Production

Norovirus ORF2 sequences were synthesized by Bio Basic (Amherst, NY, USA), and VLPs were expressed in baby hamster kidney cells, using Venezuelan equine encephalitis virus replicons expressing norovirus ORF2, as described previously [43]. Particle integrity was confirmed by visualization of particles approximately 40 nm in diameter, using electron microscopy. Accession numbers for the genotypes used to make VLPs for the blockade screening are the following: GI.1-M87661, GI.3-JQ743330.1, GI.4-JQ743331.1, GI.5-KJ402295.1, GI.7-AEQ77282.1, GI.6-AB081723.2, GII.2-MT767367.1, GII.3-JQ743333.1, GII.4 Sydney 2012- AGJ52172.1, GII.12-KP64099.1, GII.14-AY130761.1, GII.17-KP698930.1, GII.4 Den Haag 2006-JQ478409.1.

### 2.7. Antibody Blockade of VLP Binding Assay

In brief, VLPs (0.25 μg/mL) were pretreated with 10-fold serial dilutions of serum for 1 h at 37 °C, transferred to wells coated with pig gastric mucin type III (Sigma-Aldrich, St. Louis, MO, USA) or human type B saliva (GII.2, GII.12 VLP only) and incubated for 1 h at 37 °C. Bound VLP was detected, as described previously [44], using anti-VLP rabbit hyperimmune sera. All sera were screened against each VLP. Any sample with detectable titer was repeated. A positive control was included on each plate. All reactions were performed in duplicates. Percent control binding was calculated using the following formula: % Control binding = (OD VLP + serum/OD VLP) × 100. A sigmoidal dose-response model was fit to the blockade data in GraphPad Prism 9.1.2. Regarding inhibitory dilutions, 50% (ID50) were calculated for sera that demonstrated blockade of at least 50% at the lowest dilution tested. Sera that did not block 50% of binding at the lowest dilution tested were assigned an ID50 of half the assay lower limit of detection for statistical comparison [44].

### 2.8. Statistical Analysis

Statistical analyses were performed using GraphPad Prism 9.1.2 [25,28]. ID50 values were log transformed for analysis. The correlation between fold increase-paired VLP was determined by the Spearman’s rank correlation coefficient test. Seroconversion was defined as a post-infection ID50/pre-infection ID50 ratio > 4. Duration of Ab immunity post-infection was evaluated using the Mann–Whitney test. A difference was considered significant if *p* < 0.05.

## 3. Results

### 3.1. Genetic Diversity of Norovirus in Children < 2 Years of Age in the Nicaraguan Birth Cohort 

**Figure 1:**
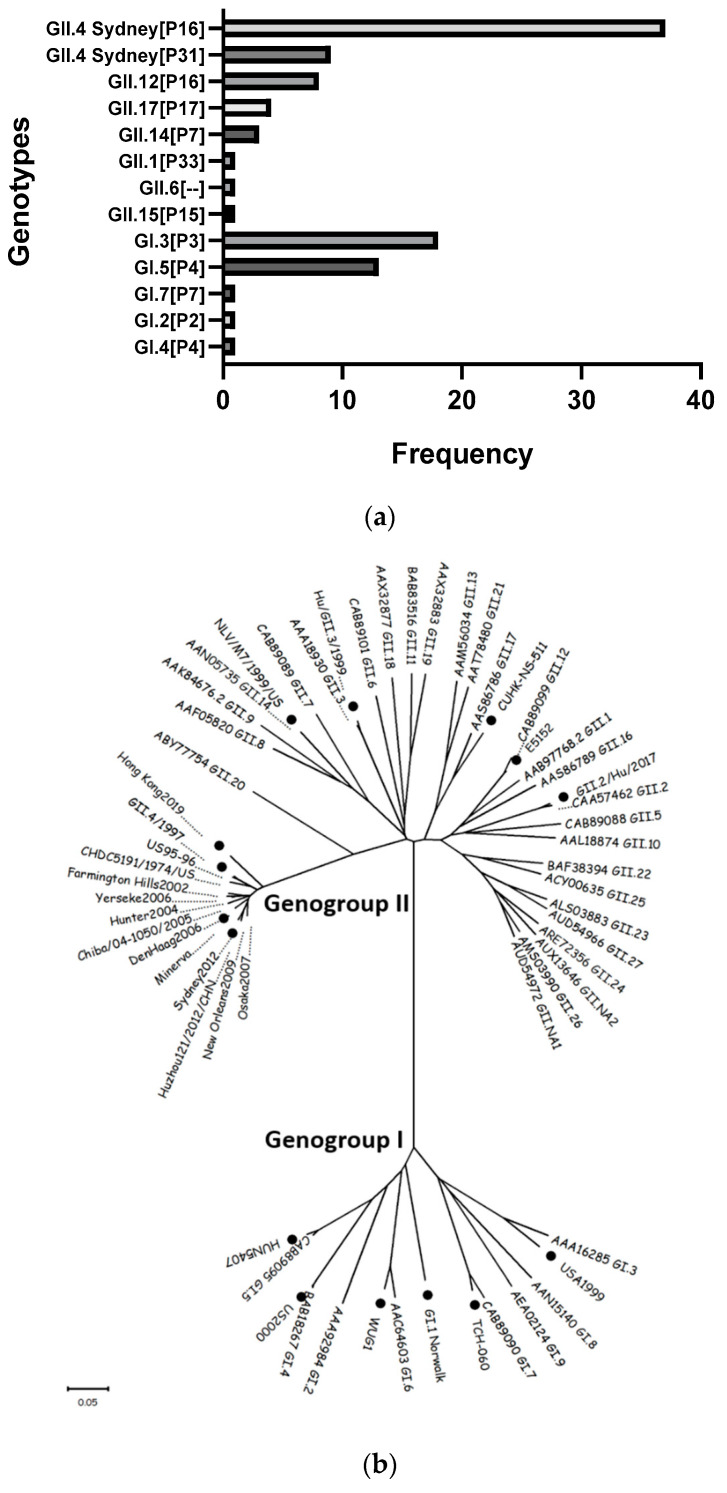
(**a**) Genetic diversity of the noroviruses circulating in children < 2 years of age participating in a Nicaraguan birth cohort. Genotypes identified are based on the capsid followed by the polymerase gene (RdRp); (**b**) Phylogenetic analysis of the VP1 amino acid sequence from reference genotypes, including the 13 norovirus genotypes and GII.4 variants selected to generate viral-like particles (VLPs) to be used as antigens in the blockade assay (black dots). These VLPs are representative of the most common genotypes circulating in the birth cohort during the surveillance period.

**Figure 2:**
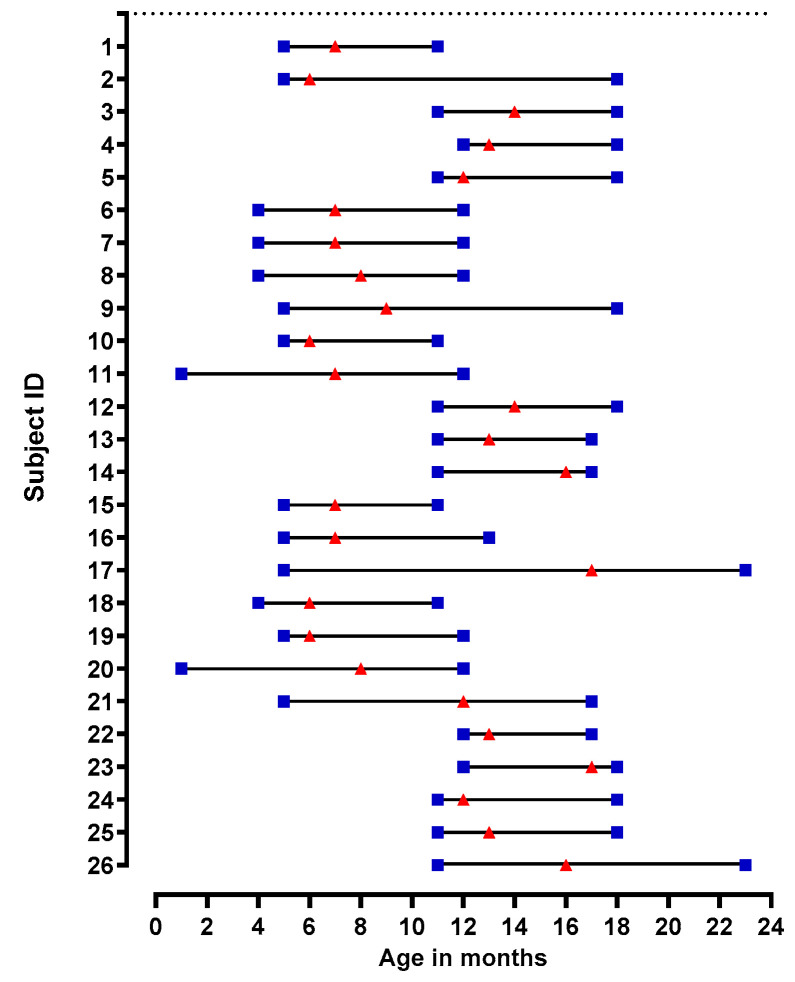
Age of the children at pre- and post-serum sampling and age at first norovirus AGE episode. Blue squares represent the age at pre- and post-sampling. Red triangles represent age at the first norovirus AGE episode. Children 1 to 14 experienced monotypic blockade responses and children 15 to 26 experienced multitypic blockade responses.

Of the 1031 AGE episodes reported in the birth cohort between June 2017 and June 2019, 846 (82%) stool samples were collected and analyzed. Of these, 153 (18%) were norovirus-positive, including 66 (43%) positive for GI and 87 (57%) positive for GII. The median age of first symptomatic infection was 12 months (IQR 8 to 16 months) and 60% of the cohort participants who experienced a norovirus episode were boys. Of the norovirus-positive stools, 98 (64%) were successfully genotyped, with the most common genotypes identified as GII.4 Sydney [P16] (38%), GII.4 Sydney [P31] (9%), GI.3 [P3] (19%), GI.5 [P4] (13%), GII.12 [P16] (8%), GII.17 [P17] (4%), GII.14 [P7] (3%) (Figure 1a). Of the children with known genotypes, a subset of 26 were selected according to the following criteria: (1) experienced their first norovirus episode before 18 months of age, (2) were infected with one of the four most common genotypes circulating in the cohort and (3) provided pre- and post-episode serum samples of sufficient volume. These children also provided 397 monthly stools. In the 26 children analyzed, the median age at the first norovirus AGE episode was 11 months (IQR 7 to 13) (Figure 2).

### 3.2. Norovirus Antibody Response and Pre-Existing Antibodies

To determine the breadth of blockade Abs induced after the first norovirus AGE episode, pre- and post-infection sera were tested against four VLPs representing the most common genotypes circulating in this birth cohort. Additionally, nine more VLPs were tested to evaluate potential cross-protection and possible norovirus serotypes (Figure 1a,b). While 10 (38%) of the 26 children did not show pre-existing Abs before the first AGE episode, blockade antibodies against GI, GII or both genogroups were observed in one (4%), eight (31%) and seven (27%) children, respectively. The children with pre-existing blockade Abs to both genogroups recognized between 2 and 11 different VLPs (Table 1). The median age of five months in children both with and without pre-existing blockade Abs at pre-episode sampling was not different, indicating that titers likely represent undetected asymptomatic infections, as well as residual maternal antibodies (Figure S1 and Figure 2 and Table 1).

Pre-existing blockade Ab titers may be protective against reinfection with the same genotype. In the 16 children with pre-existing titers, there were 27 instances of pre-existing titers to any GII genotype tested (light-gray boxes, Table S1), only four (14.8%) of which subsequently seroconverted to the same genotype. There were also 24 instances of pre-existing titers to GI genotype, only two (8.3%) of which subsequently seroconverted to the same genotype (light-gray boxes, Table S2). A chi square analysis showed a relative risk of GII seroconversion of 0.6 (95%CI 0.597 to 0.608, *p* = 0.005) in children with GII pre-infection titers > 50 compared with pre-infection titers < 50. Higher pre-infection titers were associated with greater protection from subsequent seroconversion. 

### 3.3. Monotypic and Multitypic Seroconversion in Children Experiencing the First Norovirus AGE Episode before 18 Months of Life

**Figure 3:**
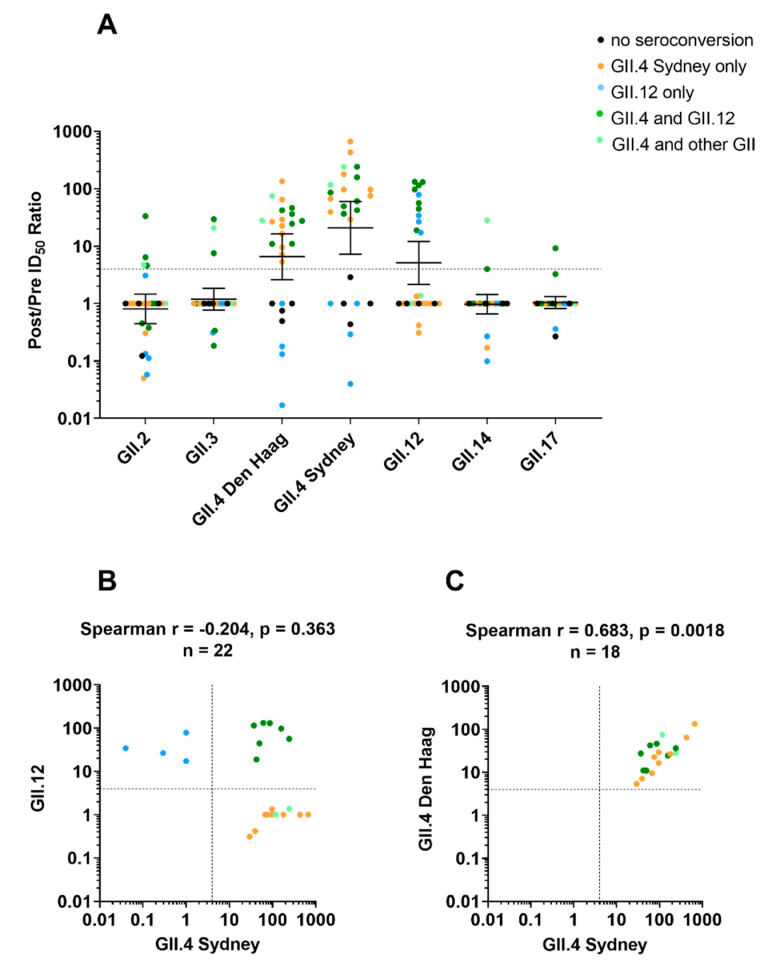
Patterns of blockade antibody seroconversion following infection with genogroup II human noroviruses. (Panel **A**): Univariate scatter plot of the fold increase in blockade antibody response between pre- and post-genogroup II norovirus infections; children color-coded by seroconverted genotype(s) identified by blockade Ab assay; dashed line indicates a ≥4-fold increase. X-axis is VLP used to test sera. Seroconversion color-coding: none (•), GII.4 (•), GII.12 (•), GII.4 and GII.12 (•) and GII.4 plus any other genogroup II genotype (•) (*n* = 26). Marker: one child. Line and error bars: geometric mean and 95% confidence intervals. Spearman’s correlation analysis of the fold increase in blockade antibody responses between GII.4 Sydney and GII.12 (Panel **B**) and between GII.4 Sydney and a closely related variant, GII.4 Den Haag (Panel **C**) among samples with 4-fold increase to one VLP. Dashed lines: 4-fold increase.

**Figure 4:**
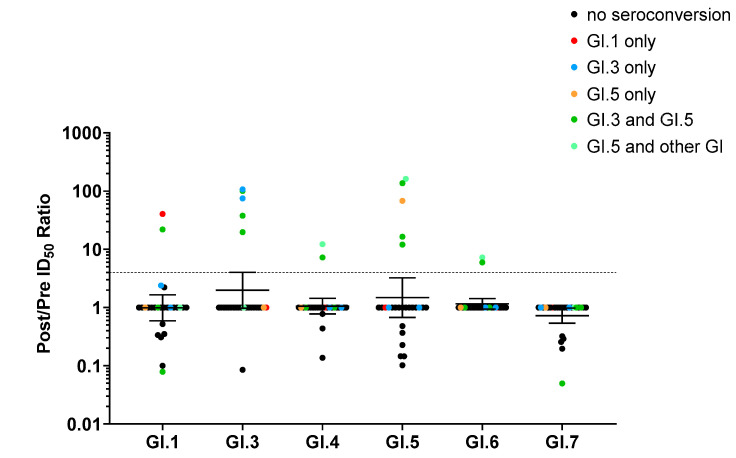
Patterns of blockade antibody seroconversion following infection with genogroup I human noroviruses. Univariate scatter plot of the fold increase in blockade antibody response between pre- and post-genogroup I norovirus infections; color-coded by the seroconverted genotypes with a ≥4-fold increase: none (•), GI.1 (•), GI.3 (•), GI.5 (•orange), GI.3 and GI.5 (•), and GI.5 plus any other genogroup I genotype (•) (*n* = 26). Marker: one child. Line and error bars: geometric mean and 95% confidence intervals. Dashed line: 4-fold increase.

Of the 26 children included in the study, there were 13 monotypic GII seroconversions (nine GII.4, median fold increase 96.8 IQR 53.2–304; and four GII.12, median fold increase 30.4 IQR 19.6–67.5). There were also four monotypic GI seroconversions (one GI.1, fold increase 40.5; two GI.3, fold increases 74.6 and 107; and one GI.5, fold increase 67.8). Three of the children had single seroconversions in one genogroup and multiple seroconversions in the other. Additionally, there were nine multitypic seroconversions that included GII.4 (median fold increase 86 IQR 45.9 to 199.5) and seven multitypic seroconversions that included GII.12 (median fold increase 97.3 IQR 44.5 to 131). There were also four multitypic GI seroconversions. Three included GI.3 titer (fold increases 19.7, 37.6, and 100.3), and all four included GI.5 (median fold increase 76.2 IQR 13.1 to 155) (Figure 3A and Figure 4, Table 1, Tables S1 and S2).

### 3.4. Correlation between Ab Blockade Seroconversion and Norovirus Genotyping

PCR-derived genotype distribution among the 26 children experiencing the first symptomatic infection, and participating in this study, was GII.4 Sydney (*n* = 15, 57.7%), GII.12 (*n* = 5, 19.2%), GI.3 (*n* = 3, 11.5%) and GI.5 (*n* = 3, 11.5%). When the genogroups were combined, 25 of the 26 (96.2%) children seroconverted to the PCR-detected infecting genotype. However, when analyzing the genogroups separately, 20 of 22 (91%) GII-seroconverted children matched their PCR-detected infecting genotype, and five of eight (62.5%) GI-seroconverted children matched their PCR-detected infecting genotype. The discrepancy of sample sizes when looking at combined genogroups vs. separated genogroups was due to the four children who seroconverted to both genogroups. 

### 3.5. Blockade Ab Response Was Genotype-Specific but Not Variant-Specific

The most frequent pairing in multitypic seroconversions was GII.4 Sydney and GII.12. Of the 18 children who seroconverted to GII.4 Sydney, seven also seroconverted to GII.12. Among children with titers to either GII.4 or GII.12, blockade antibody titers between GII.4 2012 Sydney and GII.12 did not correlate (*p* = 0.363, *r* = −0.204, *n* = 22, Spearman’s rank correlation analysis) (Figure 3B). In contrast, blockade antibody titers to GII.4 Sydney highly correlated with titers to GII.4 Den Haag (*p* = 0.002, *r* = 0.683), a related GII.4 variant (Figure 3C). The correlation between other blockade Abs response pairs was not significant, but the small number preclude any reliable conclusion.

### 3.6. Asymptomatic Norovirus Infections in Children with Multitypic Blockade Ab Response 

To better understand the role of asymptomatic norovirus infections in the multitypic blockade Ab response observed, all available monthly (asymptomatic) stools from the 26 children were tested for norovirus. Twenty-three percent (6/26) of the children had asymptomatic norovirus detected in at least one monthly stool. These infections included GI.3 and GII that could not be typed due to low viral load. Thus, symptomatic virus detection at the genogroup level modestly improved concordance between serological and molecular detection, suggesting that additional infections between the monthly intervals tested may have occurred. When comparing the PCR analysis of all the stool samples collected with the complete serology panel, 15 of 26 (57.7%) agreed when genogroups were combined, 12 of 22 (54.5%) matched among the GII-seroconverted children, and three of eight (37.5%) matched among the GI-seroconverted children (Tables S1 and S2). 

### 3.7. Duration of Norovirus Blockade Ab Titer after First AGE Episode

**Figure 5:**
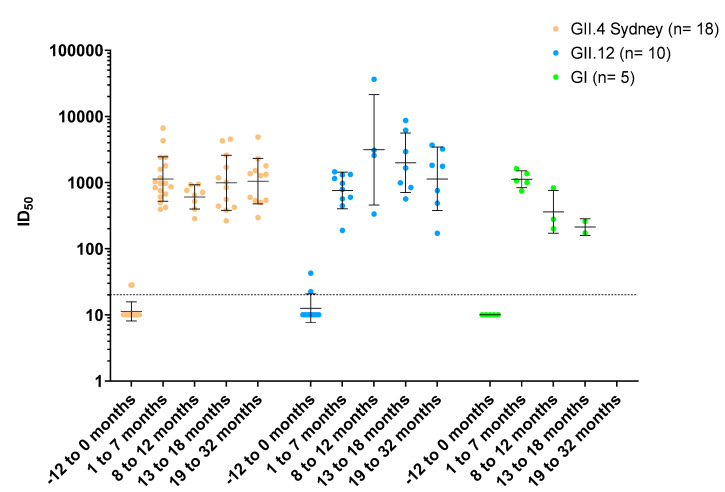
First norovirus episodes induce durable blockade antibody responses in young children. Additional serum samples from the infected children with serological data presented in Figure 3 and Figure 4 were collected over a 2-to-3-year period and analyzed for blockade antibody titer to the infecting genotype: GII.4 Sydney (•), GII.12 (•) and GI.3/GI.5 (•). Marker: one child. X axis: time period in months relative to first AGE episode. Line and error bars: geometric mean and 95% confidence intervals. Dashed line: assay limit of detection.

Twenty-three of the 26 children had additional sera collected every 6 months for up to 32 months after the AGE episode, allowing for the measurement of the duration of blockade Ab responses relative to their pre-episode serum sample. Positive sera were grouped by GII.4 Sydney, GII.12 or any GI VLP and each titer followed over time. GII.4 Sydney and GII.12 titers remained consistent for at least 19 months post-infection. The response to GI genotypes trended downward after 1 year (*p* = 0.071, Mann–Whitney), although numbers were small (Figure 5). Of these children, none experienced a second infection of the same genotype during the study period.

## 4. Discussion

The development of pediatric norovirus vaccines has been hindered by the genetic diversity of norovirus, a lack of defined norovirus serotypes and little knowledge about how immunity to norovirus develops in naïve children. Birth cohort studies provide a unique opportunity to assess the immune response to norovirus infections from birth and examine the breadth and duration of immunity induced by natural infections over time. The extensive serology data generated in this study measuring blockade Abs, a surrogate of neutralizing Abs, is useful to define possible norovirus serotypes and to characterize natural immunity following first norovirus AGE episodes in young children who would likely be targeted by future norovirus vaccines.

Our findings show that seroconversion following first natural episodes was primarily genotype-specific. We found monotypic seroconversion in 65% of children, suggesting that a single infection induces genotype-specific Abs that block the binding of norovirus VLP to HBGAs, and those Abs are not cross-reactive between genotypes. The monotypic humoral response observed in this study aligns with homotypic protection against re-infection reported in an observational study in Peru, in which Abs response was not investigated [46]. Immunological studies examining small numbers of children also show homotypic responses [32,47,48], but experimental studies in adults show broad responses following ingestion of one specific strain. For instance, Czako and coworkers observed that experimental infection with Norwalk virus (GI.1) in adults induces heterotypic HBGA-blocking activity against multiple genotypes, including GII.4 [49]. Similarly, a phase 1 clinical trial in adults showed that a multivalent GI.1 and GII.4 VLP-based vaccine elicited broadly blocking Ab, with activity against the norovirus genotypes not included in the vaccine [28]. To understand why some experimental and clinical trials in adults show broader humoral immunity at the genotype level than the monotypic immunity observed in most children from this sub-cohort warrants further studies on the development of the B cell repertoire following natural norovirus infections. 

We identified multitypic seroconversion in 46% of the children from this sub-cohort. The majority of multitypic seroconversions that occurred in our study included both GII.4 and GII.12, the two most common circulating genotypes observed in the cohort during the surveillance period. A correlation was not observed between GII.12 and GII.4 blockade Ab titers post-infection with either genotype, while correlation was observed in the response between different GII.4 variants. This suggests that the multitypic patterns observed reflect intervening asymptomatic infections rather than development of cross-reactive blockade Abs. In further support for this hypothesis are the differences in P2 domain aa sequences between GII.4 and GII.12, calling into question the presence of cross-reactive epitopes, while conserved epitopes have been found between GII.4 variants [44]. Indeed, most of the neutralizing and HBGA-blocking B cell epitopes have been mapped in or proximal to the surface-exposed P2 region of the P domain [50]. As our monthly stool collection schedule did not capture all of these asymptomatic infections, future studies should consider weekly stool collection for surveillance of asymptomatic norovirus infections to fully capture norovirus infection history [46]. The global prevalence of asymptomatic norovirus infection has been observed to range from 6% to 9%, with a higher prevalence in Africa (15%), Meso America (14%) and South America (11%), suggesting that multiple exposure leading to multitypic seroconversion could be a very common event [51,52]. 

Pre-existing humoral immunity correlated with reduction of developing a future AGE episode associated with the same genotype. Most of the children (62%, 16/26) had pre-existing blockade Ab titers and only five of these seroconverted to the same genotype as the blockade Ab within a range of 1 to 5 months after pre-serum sampling. Atmar et al. report that serum blockade Ab titers above 200 were associated with a 72% reduction in frequency of illness and a 57% reduction in infection, following vaccination and challenge in human volunteers, suggesting that pre-challenge blockade Ab titers correlated to protection [53]. The observation that 12 out of 16 children with preexisting Ab were not re-infected with the same genotype is suggestive of serotype-specific protection. The presence of Abs in pre-serum samples have been associated with strain-specific protection in previous studies [32,54].

The GII.4 Sydney variant was responsible for approximately 50% of the norovirus AGE episodes in this sub-cohort, which agrees with the global trends of norovirus genotype distribution among children ≤ 2 years of age with an AGE episode, as reported by the global pediatric norovirus strain surveillance network [16]. Moreover, our serological assay suggests even higher circulation of GII.4 viruses in this cohort than that detected in diarrheal stools by RT-qPCR, as most of the children examined (69%, 18/26) had an HBGA blockade antibody to the GII.4 Sydney strain. Interestingly, only 5 of the 26 children experienced a second norovirus AGE episode up to 32 months of age (GII.7, GI.3 and three GII.NT). None of these second episodes was of the same genotype, indicating that early childhood norovirus AGE episodes induce durable neutralizing Ab responses, and correlation with protection from infection. The immune mechanisms of protection warrant further exploration in available culture systems [55].

In addition to understanding the breadth of immunity following first norovirus AGE episodes, this study examined the duration of blockade Abs in the birth cohort. Blockade Ab titers remained consistent for at least 19 months post-infection. Data from early human challenge studies conducted in adults suggest short-term norovirus immunity (2 months to 2 years) [56,57] based on protection to illness after re-challenge. However, the infecting dose used in those studies was higher compared to the estimated natural infectious dose (1320 genomic equivalents) [58]; for instance, the 8fIIa Norwalk inoculum used in the first human challenge was 7.6 × 10^7^ RT-PCR detectable units [59]. Using a mathematical model based on transmission at community level, Simmons, et al. estimated that immunity might persist from 4 to 9 years [60]. Further, blockade analysis of serum collected from two children until eight years of age showed that norovirus GII.4-specific Abs with high blocking potential and avidity were developed after two years of age and were retained throughout the follow-up [36]. The findings presented in the current study provide novel and practical information on the duration of blockade Abs, a surrogate for neutralizing Abs. 

This study has some limitations. The number of children included in the analysis was low, but many studies trying to investigate serologic response over time have faced the same challenge [32,36,48]. Multitypic seroconversions could not be explained by asymptomatic infections in all cases, likely because routine samples were collected monthly and norovirus shedding can be limited to 8 days in some instances [46]. Therefore, a monthly sampling schedule would be unable to capture all asymptomatic norovirus episodes. Furthermore, asymptomatic infections with a lower viral load might not be captured. The finding of more infections detected by serology compared to by molecular detection highlights the benefit of monitoring blockade Ab titers over time to define infection history, and the high burden of both symptomatic and asymptomatic norovirus infections in early childhood.

## 5. Conclusions

In conclusion, we found genotype-specific blockade Ab responses against first norovirus AGE episodes in early childhood, in contrast to the broad response observed in experimentally infected adults. Blockade Ab responses lasted for at least 19 months after infection, suggesting durable homotypic protection. The multitypic seroconversions in which the most common response was against the circulating GII.4 variant and lack of correlation in blockade Abs between genotypes is indicative of asymptomatic infections that might broaden antibody response over time. These findings support the need for pediatric norovirus vaccines to include GII.4 as a principal component, and possibly several other genotypes (including those infecting non-secretors) [14,40] to provide broad protection in any population. It also highlights the need to administer norovirus vaccines early in life where the burden of norovirus disease is high. Finally, our study provides optimism for the duration of immunity induced following a first norovirus exposure that can potentially be replicated or expanded upon by vaccination.

## Figures and Tables

**Table 1:** Characteristics of children included in this sub-cohort and blockade antibody responses in sera collected before and after the first norovirus acute gastroenteritis episode.

Subject ID	Gender	Poverty Index *	Severity Score **	Pre-Existing Blockade Antibodies ^†^	Norovirus Genotype in Stools	GII Blockade Response ^⋆^	GI Blockade Response ^⋆^
1	M	1	4	GII.2, GI.1, GI.5	GII.4 Sydney [P31]	GII.4 Sydney	-
2	F	2	4	-	GII.4 Sydney [P16]	GII.4 Sydney	-
3	M	1	7	-	GII.4 Sydney [P16]	GII.4 Sydney	-
4	F	2	7	-	GII.4 Sydney [P16]	GII.4 Sydney	-
5	M	2	9	GII.12	GII.4 Sydney [P16]	GII.4 Sydney	-
6	M	2	8	GII.12, GI.1, GI.4, GI.5, GII.4 Den Haag, GI.7, GII.4 Sydney	GII.4 Sydney [P16]	GII.4 Sydney	-
7	F	2	9	GI.4, GII.14	GII.4 Sydney [P16]	GII.4 Sydney	-
8	F	1	8	GII.2	GII.4 Sydney [P16]	GII.4 Sydney	-
9	M	2	5	GII.2	GII.12 [P16]	GII.12	-
10	M	1	6	GII.2, GI.3, GII.14, GI.1, GI.5, GII.4 Den Haag, GI.7, GII.4 Sydney, GII.3, GII.17, GI.4, GII.12	GII.12 [P16]	GII.12	-
11	M	1	7	GII.4 Den Haag, GII.4 Sydney, GII.2, GII.12, GI.5, GII.14, GI.1	GII.12 [P16]	GII.12	-
12	M	1	4	-	GI.3 [P3]	-	GI.3
13	M	1	8	GII.17, GII.4 Sydney, GII.4 Den Haag	GI.5 [P4]	-	GI.3
14	F	2	3	-	GI.5 [P4]	-	GI.5
15	F	1	3	-	GII.4 Sydney [P31]	GII.4 Sydney, GII.12, GII.2, GII.3, GII.17	-
16	M	1	15	-	GII.4 Sydney [P31]	GII.4 Sydney, GII.12, GII.2	-
17	M	1	8	-	GII.4 Sydney [P16]	GII.4 Sydney, GII.12, GII.2, GII.3	-
18	M	0	8	GII.2, GI.5, GI.1, GII.3, GI.7, GII.4 Den Haag, GII.4 Sydney	GII.4 Sydney [P31]	GII.4 Sydney, GII.12	-
19	M	2	4	-	GII.12 [P16] ^§^	GII.4 Sydney, GII.12	-
20	M	2	3	GI.1, GI.5, GI.7	GII.12 [P16]	GII.4 Sydney, GII.12	-
21	M	1	5	-	GII.4 Sydney [P16]	GII.4 Sydney, GII.14, GII.2	-
22	F	0	5	GII.2, GII.4 Den Haag	GI.3 [P3]	-	GI.3, GI.4, GI.5, GI.6
23	M	1	4	GI.7, GI.1, GI.3, GI.5, GII.4 Den Haag	GI.5 [P4]	GII.12	GI.3, GI.5
24	M	2	3	GII.12	GII.4 Sydney [P16]	GII.4 Sydney	GI.3, GI.4, GI.5
25	F	2	7	GII.12	GII.4 Sydney [P16]	GII.4 Sydney, GII.3	GI.1
26	M	2	4	GII.2, GII.3	GI.3 [P3]	GII.4 Sydney, GII.12	GI.3, GI.5

* Poverty index: 0, No poverty; 1, Poverty; 2, Extreme poverty. ** Episode severity score was defined using a scale of 0–15, in which points were assigned based on symptom severity (diarrhea, vomiting, maximum of stools per day, presence of fever and if they received intravenous fluid for dehydration) [37,45]. ^⋆^ Response was defined as >4-fold increase over baseline. ^†^ Ordered from highest to lowest titer. ^§^ A second symptomatic episode due to GII.4 Sydney (11 months of age) was collected within pre-and post-serum testing.

## Data Availability

The data presented in this study are available on request from the corresponding author. The data are not publicly available due to limitations in material transfer agreement.

## Supplementary Materials

The following supporting information can be downloaded at: https://www.mdpi.com/article/10.3390/v14092053/s1, Figure S1: median ages of children with no pre-titers vs. children with pre-titers; Table S1: Serology of all children against all GII VLPs; Table S2: Serology of all children against all GI VLPs.
