# Supplementary material for: Norovirus Infection in Young Nicaraguan Children Induces Durable and Genotype-Specific Antibody Immunity
Source: Viruses. 2022 Sep 16;14(9):2053. doi: 10.3390/v14092053 (PMC9501366; doi:10.3390/v14092053)
Supplement: Supplementary file 1 [file viruses-14-02053-s001.zip › viruses-1887201-supplementary.pdf]

Supplemental Data

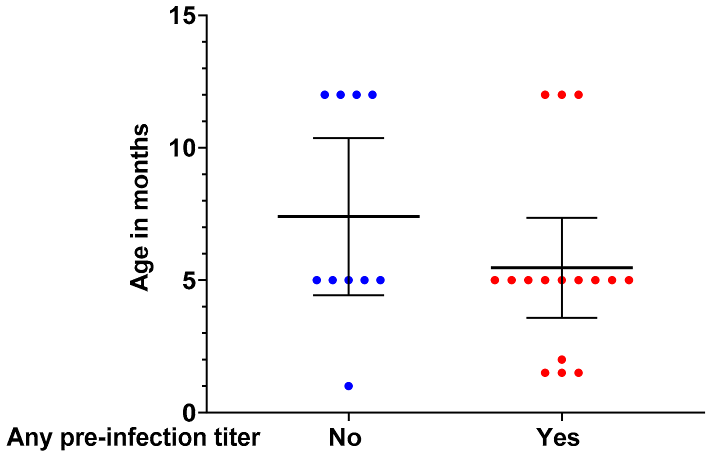

Figure S1. Age does not associate with pre-infection serum blockade antibody seroprevalence in this cohort. Children were stratified by detectable levels of pre-infection blockade antibody titer and age. Age at sample collection was not different between children with and without pre-infection blockade antibody titer (Mann-Whitney test, $p = 0.26$). Marker, one child. Line and error bars; mean and 95% CI. Y axis; age in months.

Table S1. Summary metrics to genogroup II human norovirus strains among the child cohort.

A<br>Subject number	Age at pre-blood draw months	B<br>GII.2 Pre	GII.2 Post	C<br>GII.2 Ratio	GII.3 Pre	GII.3 Post	GII.3 Ratio	GII.4 2006b Pre	GII.4 2006b Post	GII.4 2006b Ratio	GII.4 2012 Pre	GII.4 2012 Post	GII.4 2012 Ratio	GII.12 Pre	GII.12 Post	GII.12 Ratio	GII.14 Pre	GII.14 Post	GII.14 Ratio	GII.17 Pre	GII.17 Post	GII.17 Ratio	RT-qPCR	Seroconverted to infecting genotype	Serology matches asymptomatic-symptomatic PCR	Pre infection titers
1	5	199.3	10	0.05	10	10	1	10	291.6	0	10	968.4	96.84	10	10	1	10	10	1	10	10	1	Sydney	+	+	+
2	5	10	10	1	10	10	1	10	225.8	22.58	10	755.1	75.51	10	10	1	10	10	1	10	10	1	Sydney	+	+	
3	12	10	10	1	10	10	1	10	1347	134.7	10	6652	665.2	10	10	1	10	10	1	10	10	1	Sydney	+	+	
4	12	10	10	1	10	10	1	10	95.13	9.513	10	669.7	66.97	10	10	1	10	10	1	10	10	1	Sydney	+	+	
5	12	10	10	1	10	10	1	10	71.09	7.109	10	393.1	39.31	644.8	268.8	0.417	10	10	1	10	10	1	Sydney	+	+	+
6	5	10	10	1	10	10	1	36	192.5	5.347	28.31	825.3	29.15	322.9	100.4	0.311	10	10	1	10	10	1	Sydney	+	+	+
7	5	10	10	1	10	10	1	10	647.1	64.71	10	4294	429.4	10	10	1	58.41	10	0.1712	10	10	1	Sydney	+	+	+
8	5	32.91	10	0.304	10	10	1	10	266.6	26.66	10	1784	178.4	10	10	1	10	10	1	10	10	1	Sydney	+	+	+
9	5	88.94	10	0.112	10	10	1	10	10	1	10	10	1	10	786.1	78.61	10	10	1	10	10	1	GII.12	+	+	+
10	5	173.1	10	0.058	32.08	10	0.312	76.52	10	0.131	34.24	10	0.292	22.43	597.7	26.65	101.3	10	0.0987	27.71	10	0.361	GII.12	+	+	+
11	2	74.48	10	0.134	10	10	1	573.1	10	0.017	253.1	10	0.04	42.56	1455	34.19	37.22	10	0.2687	10	10	1	GII.12	+	+	+
12	12	10	10	1	10	10	1	10	10	1	10	10	1	10	10	1	10	10	1	10	10	1	GI.3			
13	12	10	10	1	10	10	1	251.1	124.9	0.497	499.1	217.7	0.436	10	10	1	10	10	1	2253	601.9	0.267	GI.5 plus			+
14	12	10	10	1	10	10	1	10	10	1	10	10	1	10	10	1	10	10	1	10	10	1	GI.5			
15	1	10	333.5	33.35	10	75.36	7.536	10	422	42.2	10	604.7	60.47	10	1323	132.3	10	39.96	3.996	10	92.09	9.209	Sydney	+		
16	5	10	64.03	6.403	10	10	1	10	463	46.3	10	860.2	86.02	10	1305	130.5	10	10	1	10	10	1	Sydney	+		
17	5	10	45.88	4.588	10	294	29.4	10	109.8	10.98	10	422.4	42.24	10	188.6	18.86	10	10	1	10	32.63	3.263	Sydney	+		
18	5	204.7	92.66	0.453	54.47	10	0.184	32.11	883.2	27.51	28.1	1030	36.65	10	1149	114.9	10	10	1	10	10	1	Sydney	+		+
19	5	10	10	1	10	10	1	10	362.9	36.29	10	2407	240.7	10	564.6	56.46	10	10	1	10	10	1	GII.12	+	+	
20	1.5	10	10	1	10	10	1	10	244.9	24.49	10	1581	158.1	10	972.8	97.28	10	10	1	10	10	1	GII.12	+		
21	5	10	46.96	4.696	10	10	1	10	747.2	74.72	10	1174	117.4	10	10	1	10	281.1	28.11	10	10	1	Sydney	+		
22	5	81.02	10	0.123	10	10	1	58.09	43.58	0.75	10	28.86	2.886	10	10	1	10	10	1	10	10	1	GI.3			+
23	1.5	10	30.86	3.086	10	10	1	56	10	0.179	10	10	1	10	172.4	17.24	10	10	1	10	10	1	GI.5			+
24	12	10	10	1	10	10	1	10	164.2	16.42	10	968.4	96.84	528.8	709.3	1.341	10	10	1	10	10	1	Sydney	+		+
25	1.5	10	10	1	10	206.2	20.62	10	279.4	27.94	10	2405	240.5	1162	1600	1.377	10	10	1	10	10	1	Sydney	+		+
26	5	91.03	34.55	0.38	29.72	10	0.336	10	109.5	10.95	10	496.1	49.61	10	445.3	44.53	10	10	1	10	10	1	GI.3			+
Totals																								20 of 22	12 of 22	15 of 26

A. Single seroconversion to GII.4 — Single seroconversion to GII.12 — Seroconversion to GII.4 and GII.12

Seroconversion to GII.4 and another GII (not GII.12) — No GII seroconversions

B. Pre-infection titer > limit of detection

C. Post/pre ID50 ratio > 4

Table S2. Summary metrics to genogroup I human norovirus strains among the child cohort.

A		B					C																	
Subject number	Age at pre-blood draw months	GI.1			GI.3			GI.4			GI.5			GI.6			GI.7			RT-qPCR	Seroconverted to infecting genotype	Serology matches asymptomatic-symptomatic PCR	Pre infection titer	
		Pre	Post	Ratio	Pre	Post	Ratio	Pre	Post	Ratio	Pre	Post	Ratio	Pre	Post	Ratio	Pre	Post	Ratio					
1	5	28	61.9	2.21	10	10	1	10	10	1	20.8	10	0.48	10	10	1	10	10	1	Sydney			+	
2	5	10	10	1	10	10	1	10	10	1	10	10	1	10	10	1	10	10	1	Sydney				
3	12	10	10	1	10	10	1	10	10	1	10	10	1	10	10	1	10	10	1	Sydney				
4	12	10	10	1	10	10	1	10	10	1	10	10	1	10	10	1	10	10	1	Sydney				
5	12	10	10	1	10	10	1	10	10	1	10	10	1	10	10	1	10	10	1	Sydney				
6	5	88.9	31.1	0.35	10	10	1	73.2	10	0.14	68.6	10	0.15	10	10	1	34.5	10	0.29	Sydney			+	
7	5	10	10	1	10	10	1	380	295	0.78	10	10	1	10	10	1	10	10	1	Sydney			+	
8	5	10	10	1	10	10	1	10	10	1	10	10	1	10	10	1	10	10	1	Sydney				
9	5	10	10	1	10	10	1	10	10	1	10	10	1	10	10	1	10	10	1	GII.12				
10	5	100	10	0.1	118	10	0.08	22.9	10	0.44	98.2	10	0.1	10	10	1	39	10	0.2566	GII.12			+	
11	2	32.5	10	0.31	10	10	1	10	10	1	44	10	0.23	10	10	1	10	10	1	GII.12			+	
12	12	10	23.9	2.39	10	1068	107	10	10	1	10	10	1	10	10	1	10	10	1	GI.3	+	+		
13	12	10	10	1	10	745.8	74.6	10	10	1	10	10	1	10	10	1	10	10	1	GI.5 +				
14	12	10	10	1	10	10	1	10	10	1	10	678.2	67.8	10	10	1	10	10	1	GI.5	+	+		
15	1	10	10	1	10	10	1	10	10	1	10	10	1	10	10	1	10	10	1	Sydney				
16	5	10	10	1	10	10	1	10	10	1	10	10	1	10	10	1	10	10	1	Sydney				
17	5	10	10	1	10	10	1	10	10	1	10	10	1	10	10	1	10	10	1	Sydney				
18	5	65.1	33.8	0.52	10	10	1	10	10	1	68.9	10	0.15	10	10	1	51.1	10	0.1957	Sydney			+	
19	5	10	10	1	10	10	1	10	10	1	10	10	1	10	10	1	10	10	1	GII.12				
20	1.5	168	56.4	0.34	10	10	1	10	10	1	64.7	23.78	0.37	10	10	1	31	10	0.3225	GII.12			+	
21	5	10	10	1	10	10	1	10	10	1	10	10	1	10	10	1	10	10	1	Sydney				
22	5	10	219	21.9	10	375.6	37.6	10	72.3	7.23	10	164.1	16.4	10	59.6	5.96	10	10	1	GI.3	+			
23	1.5	126	10	0.08	71.6	1411	19.7	10	10	1	68.7	826.3	12	10	10	1	202	10	0.0496	GI.5	+	+	+	
24	12	10	10	1	10	10	1	10	122	12.2	10	1614	161	10	72.2	7.22	10	10	1	Sydney				
25	1.5	10	405	40.5	10	10	1	10	10	1	10	10	1	10	10	1	10	10	1	Sydney				
26	5	10	10	1	10	1003	100	10	10	1	10	1361	136	10	10	1	10	10	1	GI.3	+			
																					5 of 8	3 of 8	8 Of 26	Totals

A
- No GI seroconversions (black)
- Single seroconversion to GI.3 (blue)
- Single seroconversion to GI.5 (yellow)
- GI.3 and GI.5 seroconversions (green)
- GI.5 and other GI seroconversion (light green)
- Single seroconversion to GI.1 (red)

B
- Pre infection titer > limit of detection (1:20 dilution)

C
- Post/pre ID50 ratio > 4
